# DBT-galactography: a promising tool for improving the diagnostic workup of nipple discharge

**DOI:** 10.1186/s41747-020-00170-5

**Published:** 2020-08-04

**Authors:** Marco Moschetta, Vincenzo De Ruvo, Angelica Drago, Nicoletta Troiano, Simona Paolicelli, Giuseppe Rubini, Amato Antonio Stabile Ianora, Michele Telegrafo

**Affiliations:** 1 DETO–Department of Emergency and Organ Transplantation–Breast Care Unit, Aldo Moro University of Bari Medical School, Piazza Giulio Cesare 11, 70124 Bari, Italy; 2 DIM–Interdisciplinary Department of Medicine–Section of Diagnostic Imaging, Aldo Moro University of Bari Medical School, Piazza Giulio Cesare 11, 70124 Bari, Italy

**Keywords:** Breast, Digital breast tomosynthesis, Galactography, Mammography, Nipple discharge

## Abstract

**Background:**

Our aim was to compare the diagnostic performance of digital breast tomosynthesis (DBT)-galactography with that of full-field digital (FFD)-galactography for detecting intraductal breast lesions using an intra-individual design.

**Methods:**

Forty-nine consecutive patients with spontaneous, unilateral, single-pore nipple discharge and inconclusive FFD mammography and ultrasonography underwent galactography with a “COMBO” technique combining FFD- and DBT-galactography acquisitions. Examinations were independently analysed by two breast radiologists with 10-year experience. Sensitivity, specificity, and accuracy for both FFD- and DBT-galactography were calculated having histological examinations of surgical specimens as a reference standard. Data were presented as percentages with their 95% confidence intervals (CI). McNemar test was used. Interobserver agreement was assessed by using Cohen κ test for both techniques.

**Results:**

Sensitivity was 41/43 (95%, 95% CI 84.2–99.4) for DBT-galactography and 33/43 (77%, 95% CI 61.4–88.2) for FFD-galactography (*p* = 0.008), specificity 6/6 (100%, 95% CI 54.1–100.0) for both imaging tools, accuracy 47/49 (96%, 95% CI 86.0–99.5) and 39/49 (80%, 95% CI 65.7–89.8) (*p* = 0.038), respectively. The inter-observer agreement was 0.86 for DBT-galactography and 0.78 for FFD-galactography. The AGD resulted to 1.94 ± 0.64 for the combined technique.

**Conclusion:**

DBT-galactography showed a significantly higher sensitivity and accuracy than FFD-galactography for the identification of the intraductal findings, improving the possibility of a reliable diagnosis in patients with pathologic nipple-discharge.

## Key points

- Digital breast tomosynthesis (DBT)-galactography and full-field digital (FFD)-galactography were compared in 49 patients with pathological nipple discharge and negative mammography and ultrasonography.
- DBT-galactography showed a significantly higher sensitivity (95%) than FFD-galactography (77%) for the identification of intraductal pathologic findings, without trade-off in terms of specificity (100% for both techniques).
- DBT-galactography may become an important diagnostic step in managing pathological nipple discharge.

## Background

Nipple discharge represents the third most common breast complaint after pain and lump, with a reported prevalence of 7–10%. Most nipple discharges are benign or not associated with an underlying breast disease [1–3]. On the other side, the most common causes of pathologic nipple discharge consist in benign lesions, such as intraductal papilloma and papillomatosis occurring in 48% of cases, ductal ectasia in 15–20% or malignant lesions, such as papillary carcinoma, ductal carcinoma *in situ* (DCIS) and invasive ductal carcinoma with a high variable rate ranging from 1 to 45% of patients [3–5].

The diagnostic workup of pathologic nipple discharge still remains a controversial topic, and no general consensus exists in the medical literature. Mammography and ultrasonography are the first-line imaging techniques for evaluating nipple discharge, followed by cytological analysis of the secretion material [6]. Mammographic findings have been reported to be positive in 50 to 90% of patients with malignancy and in less than 50% of patients with a benign papilloma. In fact, mammography has some limitations in demonstrating small lesions, in case of lack of microcalcifications and if lesions are completely intraductal [7]. Ultrasonography of papillary lesions typically shows a solid, oval, intra-ductal mass, often associated with duct dilatation. A cystic component is also commonly seen [1, 8, 9].

Unfortunately, these methods present some limitations in evaluating intraductal lesions; therefore, negative examinations or benign findings cannot exclude an underlying malignant disease [8, 9]. Recently, the role of unenhanced and contrast-enhanced magnetic resonance imaging (MRI) of the breast for identifying and characterising lesions associated with nipple discharge has been emphasised due to its diagnostic performance. A meta-analysis including a total of 921 patients [10] showed a pooled sensitivity for any abnormality significantly higher for MRI (92%) than for galactography (69%) associated with a significantly higher pooled specificity (76% *versus* 39%, respectively); the pooled sensitivity and specificity for cancer detection were obtained for MRI only and were 92% and 97%, respectively. However, the routine use of MRI is still not recommended in routine clinical practice of nipple discharge; it can be useful in selected cases for evaluating inconclusive mammography and US [6].

For a long time, galactography (also named “ductography” or “ductogalactography”), performed after retrograde filling of the lactiferous duct with iodinated contrast material in case of spontaneous, unilateral, single-pore nipple discharge, has been considered as the standard-of-care technique, being effective in identifying and locating breast intraductal lesions [11–14]. However, the role of galactography has remained unclear with some authors recommending this tool for evaluating nipple discharge while others considering it no longer acceptable in the modern era of breast imaging [6]. The specificity and sensitivity of this diagnostic examination is widely influenced by the overlapping of different structures on the contrast-enhanced two-dimensional mammograms.

On the other hand, digital breast tomosynthesis (DBT) has been demonstrated to have higher accuracy compared with digital mammography being able to provide sectional images from different projection angles, avoiding the lesion masking effect due to the tissue superposition [15]. DBT-galactography, *i.e*., the usage of DBT instead of standard full-field digital (FFD) mammography after retrograde filling of the lactiferous duct with iodinated contrast material, has been investigated only in a recent study evaluating only five patients with promising results [16].

The aim of our study was to evaluate the diagnostic value of DBT-galactography for detecting intraductal breast lesion in the clinical setting of pathological nipple discharge and to compare its performance with that of FFD-galactography, having the histological findings as a reference standard.

## Methods

### Population

This retrospective study was approved by the University of Bari Medical School–D.E.T.O.—Department of Emergency and Organ Transplantation on January 2019. Between January 2018 and February 2019, all consecutive patients with spontaneous, unilateral, single-pore nipple discharge and negative breast ultrasonography were enrolled and underwent both FFD and DBT-galactography. Written informed consent was obtained from all patients according to the Declaration of Helsinki principles.

Patients with nipple retraction or a history of a prior nipple surgery were excluded from the study. Also patients with US findings suggesting intraductal lesions and eligible for US-guided breast biopsy (*n* = 21) were excluded from the study and did not undergo galactography.

In all cases, cytological analysis of nipple discharge material was performed. The breast was gently massaged in the direction of the nipple, and glass slides were touched to the secreted drops. The slides were then fixed by immersion in 95% ethyl alcohol and stained with a Papanicolaou stain. A pathologist with a 10-year experience in the field of breast cytology analysed all cases searching for benign (regular ductal cells, foamy cells, inflammatory cells, red blood cells) or malignant findings (clusters and single-enlarged ductal cells, nuclear pleomorphism, stripped nuclei, nucleoli, necrotic debris). In case of imaging and cytology positivity for papillary lesions or cancer, patients were referred to surgical treatment. In case of negativity, patients were invited to undergo breast MRI in order to exclude any underlying disease.

### Imaging technique

Galactography was performed by accessing the excretory duct with a dedicated 30-gauge cannula (HS, Hospital Service, Rome, Italy) and injecting a nonionic iodinated contrast agent (Iopamidol, 300 mg/mL, Bracco Imaging, Milan, Italy) up to a maximum of 1–1.5 mL. After contrast agent administration, each patient underwent FFD mammography and DBT in the craniocaudal, mediolateral oblique, and lateromedial projections during a single breast compression, using the “COMBO” technique (Giotto Tomo, Internazionale Medico Scientifica, Bologna, Italy) consisting of combined acquisition of DBT and FFD mammograms (scanning angle was 40°, step and shoot technique, 13 exposures with reduced optimised dose, pixel size of 86 μm).

### Image analysis

DBT- and FFD-galactography findings were analysed and classified by two independent radiologists with a 10-year breast imaging experience. The two readers evaluated FFD-galactograms in a first reading session and DBT images in a second session after 3 weeks, in randomised order, in order to avoid any reading influence. On both DBT and FFD-galactography, all examinations showing filling defects, filling stops or ductal distortion were classified as positive.

### Statistical analysis

Patient population was assessed by summary statistics, and the Kolmogorov-Smirnov test for normal distribution was used in order to test age distribution for normality. Sensitivity, specificity and diagnostic accuracy for FFD and DBT-galactography were calculated having post-surgical histological examination as the reference standard. The obtained performance values were reported as percentages with their 95% confidence intervals and compared by using the McNemar test. Interobserver agreement was assessed by using Cohen’s κ. All calculations were performed using the NCSS 2007 software (NCSS, LLC-Kaysville, UT, USA).

## Results

A total of 49 patients (age range, 39–73 years; normal age distribution, *p* = 0.552; mean age ± standard deviation (SD), 50.8 ± 7.8 years) with spontaneous, unilateral, single-pore nipple discharge (haematic, *n* = 29; sero-haematic, *n* = 20) were enrolled. Forty-three patients (88%) were referred to surgical treatment basing on positive imaging findings and nipple discharge cytology. In six cases (12%), any underlying papillary disease was excluded basing on both imaging findings and cytology.

**Fig. 1:**
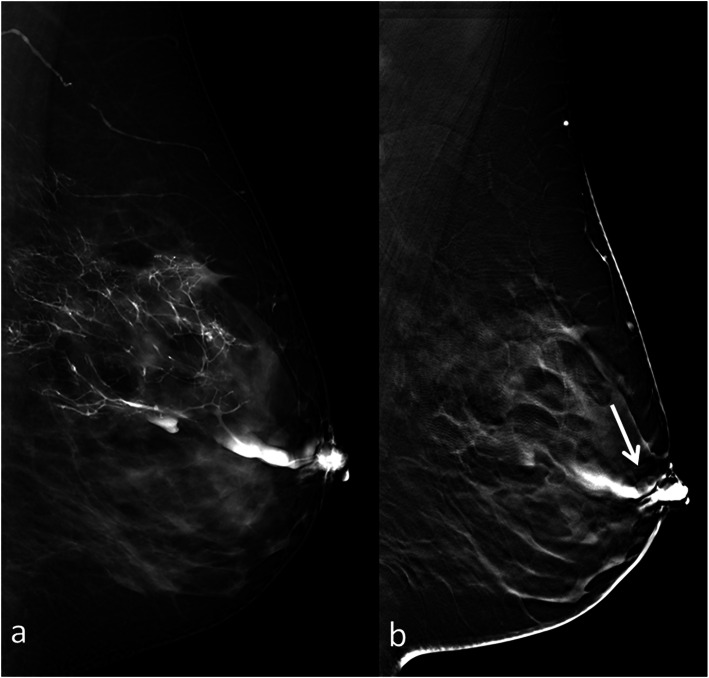
**a** Full-field digital (FFD)-galactography. **b** Digital breast tomosynthesis (DBT)-galactography. Intraductal papilloma of the left breast in a 43-year-old patient. The intraductal lesion was not identified on FFD-galactography. Thin section DBT-galactography allows the identification of the intraductal papilloma detectable as a small filling defect (arrow)

**Fig. 2:**
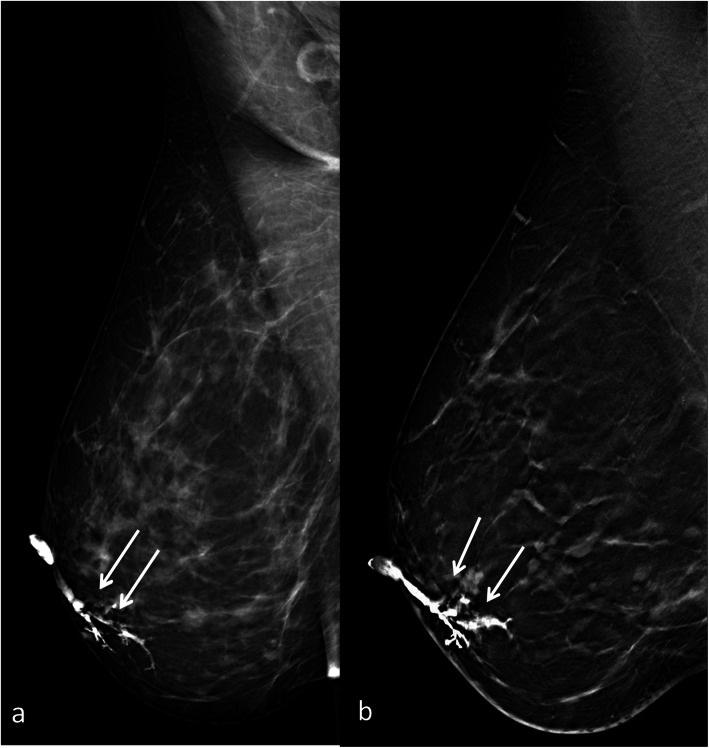
**a** Full-field digital (FFD)-galactography. **b** Digital breast tomosynthesis (DBT)-galactography. Papillomatosis of the right breast in a 42-year-old patient. The intraductal multiple filling defects are identified on both conventional and DBT-galactography (arrows)

FFD-galactography was negative in 16/49 (33%) patients (Fig. 1a) while intraductal lesions were detected in 33/49 (67%) patients; in particular, 26 (79%) single intraductal filling defects, 4 (12%) multiple filling defects (Fig. 2a) and 3 (9%) filling defects with architectural distortions were found. DBT-galactography was negative in 8/49 (16%) patients while intraductal lesions were detected in 41/49 (84%) patients; in particular, 32 (78%) single intraductal filling defects (Fig. 1b), 5 (12%) multiple filling defects (Fig. 2b) and 4 (10%) filling defects with architectural distortions were found. Nipple discharge cytology was positive in 43/49 (88%) cases and negative in the remaining 6. In particular, foamy cells, inflammatory cells, red blood cells, clusters and single enlarged ductal cells were found in 39 cases while clusters and single enlarged ductal cells with nuclear pleomorphism, stripped nuclei, nucleoli and necrotic debris in the remaining 4. All the negative patients on DBT-galactography (*n* = 8) underwent breast MRI in order to exclude any pathologic condition, and a mass-like enhancement highly suggestive for intraductal papilloma was found in 2 out of 8 patients, with positive cytology in both cases. All patients with pathologic findings (*n* = 43) underwent retroareolar duct excision, and definitive histological examination revealed intraductal disease in all cases: 18/43 (42%) single intraductal papillomas without atypia, 16/43 (37%) single intraductal papillomas with atypia, 5/43 (12%) papillomatosis without atypia and 4/43 (9%) *in situ* papillary carcinomas.

Therefore, 41 true positives, 6 true negatives, no false positives and 2 false negatives were found for DBT-galactography; 33 true positives, 6 true negatives, no false positives and 10 false negatives were found for FFD-galactography. Sensitivity, specificity and diagnostic accuracy values of 95%, 100% and 96%were obtained for DBT-galactography, 77%, 100% and 80% for FFD-galactography, respectively. In Table 1, these values are reported together with their 95% confidence intervals. Statistical analysis (McNemar test) showed a significant difference in favour of DBT for sensitivity (*p* = 0.008) and accuracy (*p* = 0.038), with the same specificity values for both the imaging tools.

**Table 1:** Diagnostic performance of digital breast tomosynthesis (DBT)-galactography and full-field digital (FFD)- galactography in 49 patients with pathologic nipple discharge

	TP	TN	FP	FN	Sensitivity (%)		Specificity (%)		Accuracy (%)	
					Point estimate	95% CI	Point estimate	95% CI	Point estimate	95% CI
DBT-galactography	41	6	0	2	95.3	84.2–99.4	100.0	54.1–100.0	95.9	86.0–99.5
FFD-galactography	33	6	0	10	76.7	61.4–88.2	100.0	54.1–100.0	79.6	65.7–89.8

*CI* Confidence interval, *FN* False negatives, *FP* False negatives, *TN* True negatives, *TP* True positives

An inter-observer agreement of *κ* = 0.86 was found for DBT-galactography and of *κ* = 0.78 for FFD-galactography.

## Discussion

Pathologic nipple discharge is typically unilateral, single-pore, spontaneous and persistent, bloody or occurring during postmenopausal phase. According to the patient age, clinical examination with mammography, US and cytological examination of the nipple discharge should be the first line diagnostic approaches. However, no general consensus exists in the medical literature, and cytological examination of nipple discharge has not been found to provide any significant complementary value [1, 6]. In case of inconclusive mammographic or ultrasonography findings, a second line examination should be considered [17].

Istomin et al. [6] recently reported that galactography is not an obsolete investigation for evaluating pathologic nipple discharge, and it remains a practical, valuable and cost-effective procedure. Conventional FFD-galactography can still represent a useful diagnostic procedure for evaluating patients with pathologic nipple discharge, since it can provide the location of the underlying lesion when a single duct discharge is identified, in contrast with cytology that can be falsely negative in a high proportion of cases [6, 18] and is also unable to provide information on lesion location. Notably, data regarding lesion location is very important for surgical planning. In this field, it has been reported that galactography lesion localisation improves the diagnostic performance of surgical biopsy from the 67% in non-studied patients to 99% in patients receiving a galactogram [18].

The galactogram may show normal findings as well as the presence of ductal dilatation, single or multiple filling defects or filling stops. In particular, suspicious findings on galactogram include distortion and ductal wall irregularity highly suggestive of malignancy, even if it is quite challenging to distinguish between malignant and benign lesions only relying on a galactogram [19].

However, FFD-galactography provide only two-dimensional images, not always allowing for accurate detection of an intraductal lesion due to overlapping effect and for precise lesion localisation in the three-dimensional space. Schulz-Wendtland et al. [16] recently reported a first approach with DBT-galactography (“galactomosynthesis”) in only five patients concluding that this kind of imaging tool could be a useful addition to complementary breast diagnostics and could lead to a renaissance of this method. However, their preliminary findings needed to be confirmed in a larger patient population comparing DBT-galactography with FFD-galactography with intra-individual design.

To the best of our knowledge, this is the first study comparing DBT-galactography with FFD-galactography in patients with pathologic nipple discharge. By assessing 49 patients with pathological nipple discharge, we found a significantly higher sensitivity and accuracy of DBT-galactography (95% and 96%) than those of FFD-galactography (77% and 80%, respectively) without any trade-off on terms of sensitivity (100% for both techniques). In other words, the gain in accuracy was entirely due to a higher sensitivity.

Our study showed that DBT galactography could represent an accurate tool for identifying and localising intraductal lesions being the cause of pathologic nipple discharge, notably also characterised by a higher inter-observer agreement as compared with FFD-galactography (0.86 *versus* 0.78).

From a general viewpoint, our results confirm the role of DBT in breast imaging, even though further studies of DBT-galactography on larger patient population are still required.

Although DBT-galactography could represent a fast, quite inexpensive, widely available and accurate examination in the field of pathologic nipple discharge, it still remains an invasive procedure to be performed only in case of nipple discharge at the time of the examination and could cause discomfort and pain [1]. On the other side, DBT-galactography uses the same conventional projections of FFD-galactography without significant technical difference. In case of validation on larger series, DBT-galactography could replace FFD-galactography in the workflow of patients with nipple discharge.

Importantly, DBT-galactography could not allow an accurate differential diagnosis between benign or malignant papillary lesions. In this field, MRI provides details in addition to the morphological information of DBT-galactography with a higher potential for lesion characterisation basing on the enhancement features [1, 10, 20]. In fact, MRI allows an accurate imaging of both breasts detecting multifocal, multicentre or occult lesions evaluating at the same time all the ductal system also in the deepest areas of the breast as compared with galactography which allows to study only segmental ductal areas. MRI has also been reported as an accurate tool for the management of nipple discharge especially in case of negative or inconclusive mammography or for a correct preoperative evaluation; despite the higher costs, it allows to exclude malignant lesions with high accuracy and to avoid unnecessary surgical procedures with a crucial role for clinical management [20, 21]. Of note, in our study, MRI has been considered as the next step in case of patients with negative findings on both FFD- and DBT-galactography. However, further studies comparing unenhanced plus contrast-enhanced MRI with DBT-galactography are needed. Finally, the recent introduction of contrast-enhanced mammography [10] needs to be investigated also in patients with pathological nipple discharge to allow for diagnosing benign, borderline or malignant lesions associated with this clinical condition.

We have also to consider that contraindications do exist for DBT-galactography mainly represented by severe allergy to iodinated contrast material, nipple retraction or a history of a prior nipple surgery that would invalidate the examination, as is for conventional galactography [1].

Our study has limitations. First, we did not evaluated differences in terms of average glandular dose (AGD) between the two techniques. This was due to the fact that we used the COMBO mode in which the two imaging techniques are acquired in a combined fashion that prevented a separation of the two doses. However, we can consider that entire AGD was 1.94 ± 0.64. This AGD, even all attributed to the DBT, means that it still remains acceptable for the clinical use, and in case of validation on larger series, the use of DBT-galactography with synthetically reconstructed images will certainly reduce the previous AVG value. Second, we studied a relatively small number of patients that, however, allowed to show a significant difference in terms of sensitivity and accuracy in favour of DBT, but not to get information about possible false positive cases that we did not encounter in our case series for both techniques.

In conclusion, we showed that DBT-galactography provided a sensitivity and a diagnostic accuracy higher than that of FFD-galactography. It could represent a reliable and largely available diagnostic tool in patients with pathologic nipple discharge, potentially avoiding the need for MRI in this clinical setting.

## Data Availability

The relevant data have been included in the manuscript. The datasets used and/or analysed during the current study are available from the corresponding author on reasonable request.

## Abbreviations

AGD: Average glandular dose
DBT: Digital breast tomosynthesis
FFD: Full-field digital
MRI: Magnetic resonance imaging
